# The autonomic nervous system and the brainstem: A fundamental role or the background actors for consciousness generation? Hypothesis, evidence, and future directions for rehabilitation and theoretical approaches

**DOI:** 10.1002/brb3.1474

**Published:** 2019-11-29

**Authors:** Davide Sattin, Matilde Leonardi, Mario Picozzi

**Affiliations:** ^1^ Neurology, Public Health, Disability Unit and Coma Research Centre Fondazione IRCCS Istituto Neurologico C.Besta Milan Italy; ^2^ Experimental Medicine and Medical Humanities‐PhD Program Biotechnology and Life Sciences Department and Center for Clinical Ethics Insubria University Varese Italy; ^3^ Biotechnology and Life Sciences Department and Center for Clinical Ethics Insubria University Varese Italy

**Keywords:** autonomic nervous system, brainstem, consciousness, consciousness disorders, review literature as topic

## Abstract

**Introduction:**

One of the hardest challenges of the third century is to develop theories that could joint different results for a global explanation of human consciousness. Some important theories have been proposed, trying to explain the emergence of consciousness as the result of different progressive changes in the elaboration of information during brain processing, giving particular attention to the thalamocortical system.

**Methods:**

In this article, a summary review of results that highlighted as cerebral cortex could not be so fundamental for consciousness generation is proposed. In detail, three topics were analyzed: (a) studies using experimental approach (manipulating stimuli or brain areas), such as decorticated animals or subliminal presentation of stimuli; (b) studies using anatomo‐clinical method (conscious inferenced from observed behaviors); and (c) data from neurostimulation of subcortical areas or of the autonomic nervous system.

**Results:**

We sketch two speculative hypothesis relative, firstly, to the possible independence from cortical areas of the on/off mechanism for consciousness generation and, secondly, to the possible role of information variability generated by the bottom‐up exchange of information among neural systems as a switch for consciousness.

**Conclusions:**

A broad range of evidence regarding the functional role of the brainstem and autonomic nervous system is reviewed for its bearing on a future hypothesis regarding the generation of consciousness experience.

## Graphical abstract

The complex role of some extra‐cortex systems, like brainstem nuclei and autonomic nervous system, should be carefully studied for consciousness generation; Brainstem presents a complex architecture with a lot of nuclei and several long and fast ways of communication between body and brain; 3‐consciousness could be an on/off mechanism.

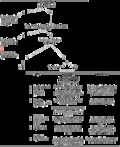

## 1 INTRODUCTION

### 1.1 Why is consciousness an open challenge so important for scientists?

Speaking about consciousness means linking together knowledge from different areas of expertise like, medicine, philosophy, neuroscience, physics, mathematics, biology, ethics, and psychology. All of these disciplines have been provided evidences derived from different hypothesis on consciousness, and the hard challenge of the third century is to develop theories that could joint different results for a global explanation of human consciousness. Why is it important? A simple answer could be because knowing if and how conscious generation is linked to matter would resolve part of the ancientest problem of the science: how our mind is linked to the body. The amount of information generated only in the last 25 years is huge on this topic: Simply typing “consciousness” as a Mesh term in the Medline database, we found that the articles published from the 3‐year periods 1994–1996 to 2015–2017 were doubled, passing from 146 to 308 papers/year. However, this amount of information did not reach common solutions to the problem cited above, and the number of theories has increased a lot in the last years as well as the definitions of what scientists refer to using the term consciousness.

Indeed, several authors refer to consciousness defining it as “the direct observation of conscious events,” as “a collective term that refers to the subjective character of our mental states, our ability to experience or to feel,” as “an interchangeably term for the mind” (Georgiev, 2013), or as “the presence or absence of experienced phenomena ‐ when one is conscious there is phenomenal content present as one is conscious of something” (Baronett, 2016; O'Doherty, 2013). Other authors go over the definition of consciousness developing new conceptualization of consciousness itself. One of the consequence of these actions was the differentiation between different forms of consciousness: from the basic ones (e.g., the experience of a smell, a scratch, or an itch) also called “primary consciousness” (Edelman, 2001), or the “phenomenal consciousness” (Block, 2007), or “phenomenal states” and “subjective experience” (Tye, 2015) to the highest ones, such as the “extended consciousness” (Damasio, 1998), implementing a new generation of different theories on consciousness.

For example, the Global workspace theory (GWT; Baars & Alonzi, 2018; Baars & Franklin, 2007; Newman, Baars, & Cho, 1997), as well as the information integration theory (IIT; Oizumi, Albantakis, & Tononi, 2014; Tononi, 2004, 2008), try to explain the emergence of consciousness as the result of different progressive changes of information among cortical areas in a process that goes toward the complexity of the information analysis.

The majority of the theories on consciousness, consider the role of the cortex fundamental for consciousness generation. The central role of the thalamocortical system in the generation of consciousness seems to be supported by several results using brain imaging techniques, for example, fMRI (Sinitsyn et al., 2018; Threlkeld et al., 2018) or magnetoencephalography (Andersen, Pedersen, Sandberg, & Overgaard, 2016; Hales, 2014). Moreover, other results supported the idea that lesions in the thalamus or in the cortex, or interruption of their normal activities through various techniques, can modify the contents of consciousness (Bogen, 1997).

However, all these results did not resolve some of the main problems related to consciousness like the hard problem of consciousness, and the scientific debate remains controversial on the real neural correlates of consciousness. Indeed, the hard problem of consciousness is the problem of explaining how and why sentient organisms have experiences, that is, how and why it is that some internal states are felt states, such as heat or pain (Chalmers, 1998), rather than unfelt states. Using other words, the problem of explaining why any physical state is conscious rather than unconscious or why a physical state is related to that conscious experience.

So, in this paper we want to proceed using a step by step method developing a consequential reasoning that should be taken into account for future theoretical hypothesis on consciousness.

### 1.2 Are we sure that the thalamocortical system is the only matter involves for the consciousness appearance?

Despite the information reported above, the same anatomo‐clinical approach cited in the last paragraph revealed that large lesions of cortex (e.g., frontal cortex [Stookey, Scarff, & Teitelbaum, 1941], even lesions of an entire cerebral hemisphere [Ameli, 1980; Sebastianelli, Saltuari, & Nardone, 2017], or severe bilateral lesions of thalamus [Rodriguez & Lee, 2013], as well as by temporal changes of the gray matter as demonstrated by the well‐known case of the white collar‐Brain [Feuillet, Dufour, & Pelletier, 2007]) were not necessarily followed by a loss of consciousness or by an unreachable of conscious experiences.

Considering the last case of the white collar, one can argue that the functions of the cortex were maintained despite the liquor dysfunction. Analyzing all data available on these cases, it is normal that the scientific communities try, for example, to explain behavioral evidences argumenting that (a) the lesions described did not damage the key brain areas for the consciousness generation and that (b) consciousness emerges from different brain areas but it is not effectively located in one of them. All of these concerns are right for us.

However, there is another criticism, that we consider particularly important, expressed by the following question that is the basic issue on which this article refers to: (c) Are there other neural circuits involved in consciousness generation?

The last point has been investigated pioneered by some authors (Fiset et al., 1999; Merker, 2005; Morsella, 2003) who tried to emphasize the role of other structures of the nervous system such as the brainstem and the autonomic nervous system (ANS). Their idea was primarily supported by a phylogenetic consideration: the total number of neurons reporting a mean range between 10 and 20 billion neurons in the human cortex and about 65–70 billion neurons in the cerebellar area. The total number of these neurons is enormous in contrast of the brainstem, diencephalon, and striatum numbers that contains only about 700 million neurons and about 6.6–7.7 billion non‐neuronal cells. However, it took several millions of years to generate the basic plan for the brainstem from the beginnings of central nervous system evolution, while the development of the cortex has exploded in the comparably brief span of 30–50 million years, with much of that taking place in <10 million years (von Bartheld, Bahney, & Herculano‐Houzel, 2016).

It was possible that consciousness did not exist before the development of the neocortex.

A multitude of answers could be hypothesized for this question, from the “consciousness exists and our experiences were mainly regulated by the other structures” one to the “consciousness exists before the neocortex but the development of the telencephalic structures assumed progressively functions that were previously of other nervous (and maybe non‐nervous) structures” one. Considering the wide range of hypothesis that could be made, the need for a conceptual framework and a methodological approach are required to explore results derived from different disciplines that could help the reasoning on consciousness generation.

Taking into account the two last questions, our article aims to focus on the possible role of the brainstem and the autonomic nervous system (ANS) for consciousness. In the present article, we present a summary review about three topics related to consciousness, reporting the results found in the scientific literature on human and animal samples. Finally, we suggested two hypotheses for future research: one on the nature of consciousness and one on the role of brainstem and ANS in its generation.

## 2 METHODS

This review is organized following a step by step process.

**Figure 1:**
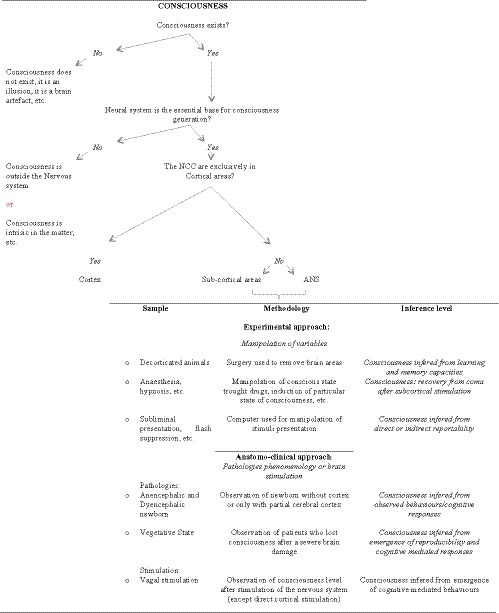
Methodological approach used for the narrative review

First of all, in the Figure 1 we presented the logical development of the manuscript, highlighting three main theoretical questions (step A). The last of them was “The Neural correlations of consciousness are exclusively in the cortical areas?,” and we then proceeded in the step B and C as the reply to this question was “No.”

In step B, we analyzed the *results from experimental research* (experimental approach) presenting direct or indirect evidences of consciousness presence in animal or human samples after manipulation of some cortical areas or some stimuli presentations in experimental settings, such as complete/almost complete cortical areas removal (surgical manipulation), use of drugs which determinates a variation in conscious level (pharmacological manipulation) or manipulation of the presentation of stimuli on conscious cognitive elaboration (cognitive manipulation);

In step C, we analyzed selected results derived from scientific literature describing findings from *clinical observation* (anatomo‐clinical approach) of patients with specific pathologies that (a) determined a subevolution of the cortical areas, (b) determined an absence of behavioral responses associated with a conscious elaboration of stimuli, analyzing also results on changes in patients' behavior responses after stimulation of their autonomic nervous system.

For all the topics, information about the level of inference about the consciousness presence was reported. The use of the narrative review form is chosen because it is the most useful for obtaining a broad point of view on a topic including two or three different perspectives, with a no established theory to test.

## 3 TOPIC 1: CONSCIOUSNESS AND THE EXPERIMENTAL APPROACH

As Crick and Koch wrote, in 1998 (Crick & Koch, 1998), “It is probable that at any moment some active neuronal processes in your head correlate with consciousness, while others do not: what is the difference between them?.” This was one of the fundamental research questions for the study of consciousness using experimental approach. In this work, we use the term conscious percept mainly referring when a subject shows some behavioral responses that indicate he/she experience became present to him/her. So, articles which used the verbal reportability and the behavioral observation of learning processes as outcome measures were taken into consideration in the next paragraphs.

### 3.1 Studies with animal samples

An article written more than 30 years ago, described experimental results related to behaviors of decorticated rats (Oakley, 1981). In detail, normal rats (group 1) and rats with 98.8% of neocortex surgically removed (group 2) were trained in a two‐choice discrimination apparatus for the food reward. The animals were first trained to run the apparatus with identical nonpatterned goal box doors, and then, a reversal training was used using horizontal and vertical striped panels attached to the doors for discrimination. Oakley concluded that “the two groups did not differ in the number of trials taken to complete preliminary training but the decorticates were significantly faster at running the apparatus than the normal ones.” He showed as the two groups did not show significant differences in the number of trials required to acquire the visual discrimination except for reversal of the discrimination that was more difficult for the lesioned group . Finally, the author wrote that “the preliminary training data are taken as further evidence that total decortication does not necessarily impair performance for food reward.”

Previously, Bjursten, Norrsell, and Norrsell (1976) observed behavioral repertory in seven cats bilaterally decorticated (in two stages, within 13 days after birth) and compared it with a control group. Chronogically, the behaviors of the decorticated kittens were similar to the others in the first weeks (crawling around, searching for and sucking their mothers' nipples). At the age of 1 month, they started leaving the box which was kept and were observed drinking from their mother's bowl of milk or eating her food. Then, they started to “play” like the others (i.e., “were observed to move around singly or in a group in sudden bursts of motor activity, running and leaping”). The first differences between decorticated and normal kittens' behaviors were found from the age of 1–2 months reporter the authors. They observed that decorticated kittens “sometimes responded aggressively when brought in contact with the latter during the play.” They then started to “withdraw” from their comrades and the world, a phenomenon which became more prominent with the progression of time. Later, at the age of 5 months, two cats were observed and photographed continuously when finding their way through a labyrinth. Authors noted that “the behaviour of the decorticate cats in the labyrinth was like that of an ordinary cat in the same situation, but slower; they played around and climbed the walls, but they also passed through some chambers from one small opening to the other along a straight line.” Bjursten et al. illustrated also a control experiment in a learning protocol T‐Maze: During these sessions, the cat was confronted with large black cups, at the ends of both arms of the maze. One cup contained the usual food reinforcement whereas the other one was kept clean. Under these circumstances, the cat found the cup containing food only 50% of the time. This finding indicates that the discriminatory behavior was independent of accessory, olfactory, or other cues which could have confused the issue. After a tailored procedure of training and sample testing, the same results were obtained for the decorticated cat and the control group. Finally, the cat was able to discriminate a large black (positive stimulus) from a large white (negative stimulus) cup between 80% and 100% of the time demonstrating learning capacity.

The link between conscious and learning is debate in literature because a lot of learning processes perform in a subliminal level without attention and someone could argue that learning is not a good example for the study of consciousness. We reported these studies because their protocols highlighted two kind of results: First, the performance was made in a new setting, that the rats/cats did not know before, and so it is hard to affirm that the learning process was entirely implicit without attention (the relationship between attention and consciousness is still debate but several theories suggested a strong linking between them); second, because were showed results on the natural evolution of behaviors in animals without cerebral cortex and this could be useful for the discussion that we reported in the final part of the article. However, considering alternative viewpoints for the interpretation of these results, researcher should be also taking into account that in cats and rats neocortex could be probably less important in learning than in humans due to the important role of peripheral systems (e.g., predominance of visual [in human] versus tactile [e.g., in cats] systems for moving around the proximal space), and so it is difficult to hypothesize the same links between learning results and consciousness in human and other mammals or between animals with different neocortex.

Another viewpoint related to the study of consciousness in animals is related to neuroanatomy. Butler and Cotterill (2006) compared neuroanatomy of nonhuman mammals, birds, and reptiles correlating their structure with a postulated perceptual or higher level consciousness following the theory of Cotterill (2001). He affirmed that “when the pattern of muscular movement, by which the creature will explore its surrounding, efference copy signals are dispatched around a specific internal loops, and this impinges upon the sensory receptors a fraction of second before the external emanating feedback signals arrive from the surroundings, this enables the nervous system to guide the animal's movements by what is essentially a sophisticated servomechanism in which the significance of impeding deviations between the intended and actual feedback is evaluated by the amigdala and corrected by the basal ganglia. So each planned sequence of muscular movements represent a series of questions […] and the nervous system will match the question with the anticipated answer that necessitates a mechanism of attention. It is for this reason that the brain components serving attention […]” and so the circuitry required for this high‐level conscious mechanism is indeed present both in avian and mammalian brains, although in this study authors did not specify directly the link between the attention and the consciousness.

Anesthesia is another field of medicine informative for the consciousness study in recent years. Minert and Devor (2016), reported that “the effect of localized lesioning of the brainstem mesopontine tegmentum (MPTA) in Wistar‐derived Sabra strain rats is sufficient to induce an anesthetic state virtually identical to systemic anesthesia” but that “destruction of the MPTA, or nonspecific suppression of its component neurons, does not induce coma as aRAS function remains unchanged” (Devor & Zalkind, 2001).

Another interesting result was shown by Flores and Colleagues (Flores et al., 2017). They found that propofol‐induced thalamic oscillations also showed changes associated with different behavioral states in rats. The thalamic and cortical oscillations showed significant increases in coherence in the α band at loss of consciousness. The role that brainstem could have in thalamocortical synchronization (a mechanism for propofol‐induced unconsciousness) is still debate but a new viewpoint was highlighted by Pillay, Vizuete, Liu, Juhasz, and Hudetz (2014). They tested the hypothesis that electrical stimulation of the pontine reticular nucleus (PnO) in the brainstem can augment information integration in the cerebral cortex of anesthetized rats with desflurane (concentrations: 3.5%, 4.5%, and 6%). The righting reflex, a surrogate measure of consciousness in rats (Zecharia et al., 2012), was barely presented at 3.5% and was immediately lost at 4.5%. At 6%, it was presumed that rats were unconscious. Authors aimed to investigate how PnO stimulation modulated the neuronal response to a visual stimulus in combined stimulation paradigms. They acquired for 10 min the spontaneous multichannel extracellular recording of unit activity (UA) and cortical local field potentials after the equilibration period at each desflurane concentration. This was followed by pontine reticular nucleus electrical stimulation: Electric current was delivered once (3 s‐on, 57 s‐off) every minute for 10 min. Finally, 10 more minutes of spontaneous UA and local field potentials were recorded after PnO stimulation ceased. Visual stimuli (30 flashes/min, 5 ms duration) were computer generated and delivered randomly with an interstimulus interval of 2 ± 1 s. Authors found that PnO stimulation produced electrocortical activation and simultaneously augmented information integration in both the parietal association cortex and the secondary visual area. In general, light flashes presented after PnO stimulation were able to significantly increase interaction entropy and PnO stimulation led to an increase in dynamic correlation in the parietal association. Authors affirmed that “taken together, the data suggest that the PnO may play a role in the modulation of cortical state and integration of sensory information under a moderate depth of anesthesia.” In our opinion, another hypothesis that should be tested is that PnO, and other neural structures with diffuse neural projections, could be not important as modulator or integrator of cortical information only but as a starting key for the system. As we describe better in the last section of this article, we can hypothesize that experience, and so consciousness, emerges from the integration of different information, but we can also hypothesize that a piece of single information can be linked to a primitive experience. In this case, consciousness could not emerge as integration but because coherent single information is given as, using a metaphor, the single electrical impulse produces car starting.

### 3.2 Studies in human subjects

The possibility to use reportability and other experimental approach (e.g., manipulating variables: attentional blink, in which the second of two target stimuli appearing in close succession is often not perceived or flash suppression, in which the perception of an image presented to one eye is suppressed by flashing a different image to the other eye) in humans allows several researchers to study when a stimulus became “conscious” to a subject. In this sense, most of the literature found the role of some cortex areas as fundamental. However, there is some interesting evidence that should be considered in the debate on consciousness. Observing the history of the cognitive neuroscience, the first literature published on the cognitive functions was mainly focused on discovering the real functional role of the cortex in the generation of specific cognitive functions, such as language, attention, and other functions. The idea was that the neocortex is the evolutionary brain area most recently developed in the mammalians history and that the superior functions should be linked to this area. In a second moment, the role of other brain areas, such as the cerebellum as well as subcortical areas and ANS, became most clear, highlighting as their architecture influences the exploitation of different high‐level cognitive functions too.

In line with this point of view, some articles were published recently trying to link the ANS activity, measured indirectly with different techniques, to brain functions and consciousness. Richter, Babo‐Rebelo, Schwartz, and Tallon‐Baudry (2017) have shown as “the 8% of spontaneous alpha fluctuations in the cortex were explained by gastric phase, and gastric‐alpha coupling appears to be driven by ascending signals from the stomach to brain.” The interesting ideas of these authors were that the alpha rhythm could be potentially correlated to activity of other organs. The alpha rhythm is known to exert an inhibitory influence on spike‐firing rate (Haegens, Handel, & Jensen, 2011; Haegens, Nacher, Luna, Romo, & Jensen, 2011) and has a versatile impact on perception, attention, and memory. Using another indirect measure of the ANS, one article analyzed both the heartbeats as ticking clocks that constantly send intrinsically generated ascending information up to the central nervous system (Tallon‐Baudry, Campana, Park, & Babo‐Rebelo, 2018). Authors, using classic learning paradigm (requires a subjective judgment), asked participants to fix a central bull's eye that turns red to indicate the beginning of a trial and a faint stimulus may be presented or not after a variable interval. After that, authors asked to participants to say whether they have seen a stimulus or not. Authors proposed that the combination of first‐person perspective with visual content defines the subjective experience and they “hypothesized that neural responses to heartbeats predict conscious perception by signaling a simple form of self, in particular in the default network,” accounting for the “I” in the report “I have seen the stimulus.”

This mechanism would be based on the constantly updated neural maps of the internal state of the body and create a neural subjective frame from which the first‐person experience can be reported. It is important to underline that the authors considered visceral information as a fundamental fuel for the neural subjective frame.

## 4 TOPIC 2: HIGHLIGHTS FROM CLINICAL RESEARCH

Another step of this study (aims to explore a different viewpoint from the idea of an one‐way role of cortex for consciousness) is to analyze evidence from pathological conditions which involve several areas of the human cortex. The development of neurophysiological and imaging techniques has lead to by‐pass the limits related to the primitive anatomo‐clinical approaches when the anatomo‐pathological analysis of the brain's damages was available only postmortem. Considering this premises, in the following section we do not refer to conscious as a report (e.g., “I have seen the stimulus”) but considering it as clinicians usually do, so observing behavioral responses to tailored stimuli whose elaboration require a different level of cognitive functions (if there is a cognitive‐mediated elaboration of stimuli, there is consciousness—this is the assumption used by several clinicians). In this sense, results from neuroimaging studies seem to support the idea that consciousness is more related to a brain process rather than it is located in a specific area of the brain, giving a fundamental importance to the cerebral cortex. This conclusion seems to be supported by the evidence that lesions in different cortex areas gave the same phenomenology, interpreted as the lack of responses indicating consciousness elaboration.

However, anatomo‐pathological observation implies observing the consciousness phenomena in a more complex way than this. In detail, the idea that consciousness is supported mainly by a cerebral cortex implies that a clinician should not observe responses to external stimuli‐conscious mediated‐ in humans (or animals) who are, for various reasons, without a cerebral cortex. Regarding this point, there are two dramatic and important situations involving fetus/newborns, with massive loss of cerebral hemispheric tissue during gestation, leading to the birth of a child missing most of the cerebral cortex (hydranencephaly), or completely without a cerebral cortex (anencephalic).

Children with hydranencephaly typically have severe motor disabilities of cerebral palsy type, but not to such an extent that they miss various forms of behavioral reactivity and expressive behavior. Aleman and Merker (2014) observed spontaneous or reactive behaviors made by children with hydranencephaly over time in familiar surroundings. The 108 caregivers participating completed a questionnaire with 106 questions related to behaviors, from the simple ones (e.g., “Does your child react to turning the room lights off or on?”) to the complex ones (e.g., “Does your child turn his/her head to or away from visual objects?” or “Does your child indicate if he/she recognises something or someone?”). In a very interesting way, 66% of caregivers reported that their children turn his/her head to or away from visual stimuli, 83% that children recognized the difference between a family member and a stranger and that children show an “awareness” of some objects. Regarding the emotional reactions, more than 85% of the caregivers reported that their children feel pain, smile, and cry (although these questions did not analyze the contingency emotional responses) and that the 45% reported that their children showed a strong aversion to someone or something (something they do not like to look at, particular activities/stories, smells, people, etc.).

These results could be interesting, considering that several of these responses are the same that are normally assessed by clinicians who are evaluating patient with disorders of consciousness (DOC), and both orientation to sound or object as well as contingency emotional reactions orienting clinicians toward a minimal conscious state (MCS) diagnosis. However, someone could argue that caregivers could overestimate some reflexes reactions of their children, and that, overall, hydranencephaly does not represent a complete absence of cerebral cortex which probably can mediated some very primitive conscious behaviors.

Therefore, to analyze better this point could be interesting to observe data from anencephalic fetus or newborns and from recent animal models.

Most of the studies on anencephalia reported that fetus produces only a few stereotypic movements during gestation and that newborns did not show any form of consciousness. In contrast to these results, Luyendijk and Treffers (1992), observed twelve newborn mero‐anencephalics, who survived for more than 1 day after birth. In 4 of the 12 children studied, touching or slight pressure, and also faradic stimulation of various spots of the cerebrovascular area were invariably followed by a facial expression which was judged congruently to the stimuli (a smile, a laugh or a grin) by the 78% of impartial observers.

Unfortunately, an intrinsic limit of the observational approach applied to the anencephalic newborn should be taken into account: The absence of cerebral cortex impaired also the development of the brainstem. Indeed, it appears almost completely devoid of neurons, fiber tracts, neural networks, or any evidence of primitive functional organization. This is important considering that the development of the brainstem is directly related to the development of the cortex.

Another interesting approach is to study patients in vegetative state (VS). Persons with this diagnosis, after a coma, appear to be awake but they lack any sign of awareness of themselves or their environment (Bernat, 2006; Leonardi, Sattin, & Raggi, 2013; The Multi‐Society Task Force on PVS, 1994). They normally show cycles of eye opening and closing, giving the appearance of sleep–wake cycles and have complete or partial preservation of hypothalamic and brainstem autonomic functions showing different levels of behavioral responses to external stimuli. A study by Riganello, Dolce, and Sannita (2012) investigated whether the heart rate variability (HRV) can predict the incidence of behavioral responses in VS subjects, so if the HRV analysis could show when a patient could be more “reactive” to external stimuli. HRV representing the change in the time interval between successive heartbeats and the Society for Psychophysiological Research (Berntson et al., 1997) defined it as an index useful to investigate the balance between the sympathetic and the parasympathetic activities. It contains low‐frequency (LF, 0.04–0.15 Hz) oscillations that result from both sympathetic and parasympathetic activity and high‐frequency (HF, 0.15–0.4 Hz) oscillations associated mainly with parasympathetic stimulation through the vagus nerve. In an experiment, Riganello et al. tested a group of patients with DOC administering 24 stimulus conditions (12 visual, 12 auditory) presented according to the Coma Recovery Scale guidelines (Giacino, Kalmar, & Whyte, 2004; the standard clinical assessment tool for disorders of consciousness). An electrocardiogram (EKG) was continuously recorded at rest (5 min baseline) as well as the authors can observe the HRV descriptors computed online. Two testing conditions were predetermined by authors: one indicating the highest probability of observing a response at HRV nuLF values in the 10–70 interval and peak LF values between 0.05 and 0.11 Hz (these intervals were defined as the “response” condition, whereas any time point with nuLF and peak LF values outside these ranges was considered a “no‐response” condition). They found that visual (54.8%) or auditory (55.3%) responses were significantly higher in a “response” group when tested at nuLF and pkLF descriptors within the value ranges indicative of sympathetic/parasympathetic balance than outside these ranges (12.9% and 16%, respectively).

This result could be particularly important because it seems to indicate that the ANS has a role behind the performance, as if it allow to access to something else useful for behavioral performance.

## 5 TOPIC 3: THE EFFECTS OF NEUROSTIMULATION

Neurostimulation is one of the boundary techniques linking neuroscience, physics, and neurosurgery expertise. In this sense, vagus nerve stimulation (VNS) is a neurophysiologic method that has been extensively used to treat refractory epilepsy, depression, and cognitive disorders (Yuan & Silberstein, 2016a, 2016b). The vagus nerve forms numerous connections with fibers that project to the basal forebrain, thalamus, hypothalamus, and cerebral cortex (Ansari, Chaudhri, & Al Moutaery, 2007; Frangos & Komisaruk, 2017). Regarding the neural basis of its actions, there are several hypotheses. For example, some authors hypothesized that VNS mediates its therapeutic effect by way of locus coeruleus activation and, hence, a subsequent increase in cerebral norepinephrine (NE) distribution as demonstrated in some animal models (Krahl & Clark, 2012; Roosevelt, Smith, Clough, Jensen, & Browning, 2006). Other studies highlighted the role of other neurotransmitters such as noradrenaline and orexin: It significantly affects recovery from TBI by promoting wakefulness and by inhibiting sleep. The VNS significantly increases firstly the extracellular noradrenaline levels in the hippocampus and prefrontal cortex, as well as 5‐HT levels in the dorsal raphe nucleus and, secondly, the dopamine levels in the prefrontal cortex and nucleus accumbens with a parallel mechanism of action (Mieda, 2017; Yuan & Silberstein, 2016a, 2016b). Consequently, some recent studies have shown that VNS affects the amounts of time spent awake and asleep, and it can decrease sleep duration. Moreover, some authors evaluated whether VNS could promote grades I–IV consciousness, as determined by observing sensory and motor functions (Yang & Friedman, 2017). Indeed, chronic intermittent vagus nerve stimulation in rats seemed to facilitate the recovery of both cognitive and locomotor behaviors following fluid percussion brain injury in rats (Smith et al., 2005).

Regarding the evidences derived from the application of the VNS, some studies showed results from VNS applied to patients with DOC (Corazzol et al., 2017; Hanaya et al., 2016; Shi, Flanagan, & Samadani, 2013; Yu et al., 2017) as well as in animal model (Dong & Feng, 2018) showing some encouraging effects as reported in Table 1.

**Table 1:** Results from studies using vagus nerve stimulation (VNS) and consciousness as outcome

References	Sample	Stimulation	Parameters	Methods/Behavioral results	Adverse events (only for human sample)
Dong and Feng (2018)	120 adult Sprague Dawley rats (half male and half female), weighing 250–300 g	VNS	Frequency 30 Hz; current 1.0 mA; pulse width 0.5 ms; Total stimulation time = 15 min	Four different groups (*n* = 30 each). In the control group, healthy rats received sham operation and anesthesia. In the TBI group, free‐fall drop was used to establish the model of TBI‐induced coma. In the stimulated group, rats with TBI‐induced coma were subjected to VNS. In the antagonist group, comatose rats were intra‐cerebro‐ventricularly injected with the OX1R antagonist SB334867 and received VNS. Eight of the 30 rats in the TBI group re‐awakened from coma, while in the stimulated group, 20 rats re‐wakened and 10 rats remained in a comatose state. Twelve rats in the antagonist group re‐awakened from coma	‐
Corazzol et al. (2017)	35‐year‐old male patient lying in a vegetative state for 15 years following traumatic brain injury	VNS	Stimulation was gradually increased to a maximum intensity of 1.5 mA	“After 1 month of stimulation, when intensity reached 1 mA, clinical examination revealed reproducible and consistent improvements in general arousal, sustained attention, body motility, and visual pursuit. Scores on the Coma Recovery Scale‐Revised (CRS‐R) test improved, mostly in the visual domain, as stimulation increased, from a score of 5 at baseline (last exam) to 10 at highest intensities (1.00–1.25 mA)”	“A clear wide opening of the eyes was observed for several hours after surgery. Each time stimulation intensity was increased, recurring episodes of cough were observed together with facial flushing and wide opening of the eyes”
Hanaya et al. (2016)	A girl with an SCN1A mutation and generalized tonic–clonic seizure and focal seizures with impaired consciousness	VNS	“4 years after the start of VNS: output current 1.50 mA, frequency 30 Hz, pulse width 500 ms, on‐time 7 s, and off‐time 0.5 min (duty cycle 30%)”	“In the last 12‐month period (43–54 months after the start of VNS), her generalized tonic‐clonic seizures and focal seizures with impaired consciousness were reduced by an average of 90% and 88%, respectively”	Not declared
Yu et al. (2017)	73‐year‐old female patient was hospitalized with the chief complaint of 50 days of DOC after cardiopulmonary resuscitation	taVNS	“TaVNS was applied to the patient's bilateral ear concha twice daily for 30 min each in four consecutive weeks, with an intensity of 4–6 mA, at a frequency of 20 Hz (<1 ms wave width)2”	“The patient presented a CRS‐R of 6 at baseline. Four weeks after taVNS treatment, the patient presented a CRS‐R of 13, demonstrating new behaviors in both motor and oromotor function, which was consistent with a diagnosis of MCS”	Not declared
Shi et al. (2013)	Twelve subjects age between 18 and 60, having sustained a moderate to severe traumatic brain injury	VNS implanted on the left side	“30 s pulses every 5 min at the lowest current of 0.5 milliamperes. The frequency will be set at 10 Hz and the current will be increased at 0.5 mA intervals to 2.5 mA. If no impact is seen, the frequency will be increased at 10 Hz intervals and the trial repeated with increasing current until maximal settings of 30 Hz and 2.5 mA are reached. If a patient progresses through the 18‐month trial without improvement at stimulation parameters up to 2.0 mA, surrogate consent, Institutional Review Board approval, and additional funding will be sought to continue titration up to 3.5 mA and 130 Hz, which are the maximal stimulator settings”	Results not published	‐

Note

“…” = citation from the original article.

Abbreviations: ISI, The interstimulus interval; taVNS, Transcutaneous auricular vagal nerve stimulation.

Another neuromodulation approach is represented by the spinal cord stimulation (SCS), a novel therapeutic option in the management of stroke and several low‐perfusion syndromes. In detail, SCS consist of a surgically implanted electrode in the epidural space (typically at C2‐C4) stimulating the spinal cord in a cyclic mode (intervals: 15 min on and 15 min off), without reaching the motor threshold. Different studies showed as SCS has two main effects: (a) it enhances global cerebral blood flow (CBF) and cerebral glucose metabolism by regulating the sympathetic system (Della Pepa, Fukaya, La Rocca, Zhong, & Visocchi, 2013; Mattogno et al., 2017) and (b) it has been found that it promotes the release of neurotransmitters and neuromodulators, such as dopamine and norepinephrine (but not epinephrine; Georgiopoulos et al., 2010; Kaplitt, 2013; Mattogno et al., 2017). A reduction of the sympathetic outflow during SCS has been demonstrated by a series of studies: The so‐called “reversible functional sympathectomy” can, at least partially, account as one of the possible mechanisms put in play by SCS to produce effects on cerebral hemodynamics. Myklebust, Cusick, Boerboom, Prieto, and Khan (1995) found norepinephrine levels markedly affected during SCS, confirming a sympathetic inactivation secondary to neurostimulation.

Moreover, the effect of SCS on CBF seems to be important also for its indirect effect on cognitive functions. In an important study using SPECT to quantify CBF, Hirao et al. (2011, 2006) found that “reduced CBF in several parts of the brain including the inferior parietal lobule, angular gyrus, and precunei had a high predictive value in demonstrating which patients with mild cognitive impairment (MCI) would go on to develop full‐blown Alzheimer's disease (AD).” The importance of the SCS effect on CBF is due to the fact that 15%–20% of cardiac output goes to the brain and studies have shown that the ability to autoregulate or maintain CBF declines during aging (Hirao et al., 2011).

The first study investigating the effects of SCS on behavioral performance of patients in VS reported that the 33.3% (two out of six) patients displayed clinical improvement (Funahashi et al., 1989). After few decades, the improvement rate from VS patients after using SCS has improved achieving the 54% (109 out of 201) as reported in a study realized in 2009 (Kanno et al., 2009). Then, as reported by Yamamoto et al. (2017), the “70% (seven out of 10) of the patients had recovered from MCS following SCS therapy: They were able to carry out complete functional interactive communication consistently and reliably, and/or demonstrate the functional use of two different objects.” Matogno et al. (Mattogno et al., 2017; Yamamoto et al., 2017) published a review comparing the results of different studies with spinal cord stimulation (11 studies) and deep brain stimulation (seven studies) in order to provide a framework for the future development of neurosurgical stimulation. Regarding SCS, a total of 318 patients were tested and results were found in a range between 25% and 90% (excluding single case study) in relation to the evaluation parameters (e.g., CBF, clinical assessment).

It is difficult to define a final consideration regarding the SCS technique and the results on conscious improvement in a comprehensive way yet. The effect on CBF seems to be solid but is difficult to link a macro‐metabolism effect on consciousness, which normally used a different time framework around millisecond rather than second as CBF. An interesting point of view reported that SCS stimulate directly the brainstem and in particular the ascending reticular activating system that regulate the awareness circuit (Della Pepa et al., 2013). This activation could impact directly to the arousal, incrementing (or switching on) in some way the “energy” of the cortical system toward external stimuli. Even though this explanation can be real, future studies are needed to better understand the interaction between aRAS, that is normally involved in wakefulness, and the consciousness as just highlighted several years ago by Moruzzi and Magoun (1949).

## 6 DISCUSSION

A broad range of evidence regarding the functional role of the brainstem and autonomic nervous system, ranging from experimental psychology and neurophysiology to clinical neurology, is reviewed for its bearing on a future hypothesis regarding the generation of consciousness experience. The idea is not to decrease the role of cortex for consciousness, but to spotlight the complex role of some extra‐cortex systems, like brainstem nuclei and autonomic nervous system, as systems able to generate information fundamental for the constitution of the conscious state, rather than limiting consciousness generation to the thalamocortical system alone.

Resuming, the first consideration derived from phylogeny and evolutionary biology: Brainstem presents a complex architecture with a lot of nuclei and several long and fast ways of communication between the body and the brain with billions of neural synapsis, and the evolutionary storyline bears this out. The brainstem and ANS help to regulate a lot of physiological responses needed for survival but not all of them should be considered as simple primordial responses. Could it be that the amount of information they give help survival without a role for consciousness appearance? A negative answer helps to explain the intentional behaviors exhibited by mammals after experimental decortication, as well as the evidence that children born without a cortex are limited in the use of superior cognitive functions but they could be present to experience, reacting congruously according to external stimuli and learning complex behaviors as well as remember it after several days. This last example could be a breakpoint. In other words: if a subject demonstrates learning capacities in a new setting and a possibility to remember what they learn have they the capacity to generate a “conscious” representation or not?

As a second point, our idea is that we do not have to delete from our mind that some scientific literature supports the idea that cortico‐centers system alone could be nonautonomous for the consciousness generation. Thinking more than 50 years ago by Penfield and Jasper, a cortical removal even as radical as hemispherectomy does not deprive a patient of consciousness or modified it, but rather of certain forms of information, discriminative capacities, or abilities, but not of consciousness itself (Penfield & Jasper, 1954; Zeki, 2016). Another example in this sense is represented by patients after a complete frontal lobotomy. Lobotomies provided evidence that there was something special about the prefrontal cortex for making plans, or do creative thinking and for our capacity for comprehensive forward planning which is a hallmark of human species (Posner, 1993). But a patient without frontal lobe is not unconscious or unable to be conscious of visual stimuli overall.

Another point of view for consciousness analysis in human person is determined by the experimental versus anatomo‐clinical approach. In this sense, scientific literature are referred to two different meanings of consciousness: In the experimental field, it is measured mainly by direct or indirect reportability (e.g., “I see it”) in the subjects analyzed, whereas in the clinical approach, clinicians observed the contingency of behavioral responses (e.g., “patient move his arms”) to the external situation (e.g., “only after command”). Then, clinicians hypothesized that responses should be supported by a conscious—more or less complex—elaboration of external stimuli in patients with heterogenous brain damages (Rouder & Morey, 2009). It is clear that in this case, we are referred to two different things that have in common the idea that for “measuring” consciousness, researchers need “indirect instruments.” However, both reportability than observation of behaviors in a clinical setting referred to consciousness as derived from a complex cognitive process placed in the cerebral cortex. The explosion of studies that registered or stimulate cortical areas in relation to reportability of perceived stimuli has been a wide diffusion, due to the development of technical systems able to register and/or stimulate a cortical area in a relatively easy way, giving a wide diffusion of this approach. Unfortunately, this is not possible for the brainstem and autonomic nervous system: firstly, because they present more complex activities related to the interaction of several nucleus and fibers; secondly, because the technique to measure their activities are often only indirectly, considering that direct electrophysiological registration must use very invasive techniques and it is rarely permitted. Probably, these serious limits have decreased the growth of studies focused on the role of extra‐cortical regions for consciousness, leaving a gap that should be filled up in the future.

### 6.1 What are the new questions proposed in this research? Hypothesis and new perspectives

In this section, we present two new viewpoints: one on the nature of consciousness itself and the second on the effective role of the brainstem nuclei and ANS in consciousness generating process.

#### 6.1.1 Consciousness as an on/off entity

The first question we propose is: is it possible that consciousness is an “on” or “off” entity?

Our raw question is supported by the idea that the cortex of vertebrates can be seen as a central system for the elaboration of contents, and upper brain stem system and autonomic nervous system could retain some key roles, developed throughout the evolutionary process, for the consciousness appearance. In this sense, the higher neural systems in the hierarchy help in principle an individual to open to more alternative solutions for its living and generally he is more adaptive than the primitive ones (Howes, Warren, Farmer, El‐Deredy, & Lewis, 2016; Merker, 2007). This hypothesis implies the idea that the term “level of consciousness” is not appropriate because what defines the “level” is the availability of the cognitive functions supported by the higher neural system and not the consciousness itself, which is simply present or not. We hypothesize a system able to manage information in two parallel ways: Variability in bottom‐up information (from peripheral to central nervous system) is important to switch on consciousness, whereas the thalamocortical system operates through cognitive functions (including attention) in a parallel way both when conscious is on or off. In this model, the brainstem centrality may be a consequence of its increased influence on key cortical regions, such as the areas involved in the default mode network for example. Using a similarity, we can start from a reflection on an object that we usually have like a car. The main aim of a car is to move people from a place to another one as well as the consciousness aim is to allow the matter to survive. In this sense, information from ANS and brainstem system could be the key for the car ignition (fundamental for the movement), although the electrical system of our car can provide a lot of performances able to increase our adaptation to the environment (e.g., lights on, air conditioning vent functioning, radio, that are useful when we are in a parked car e.g.,) also when the key is turn off.

#### 6.1.2 Variation of information as a possible conscious switch

The second question is: “What is the nature of the subcortical and ANS activities that are linked to consciousness?” or, in a better tailored way: if it is possible that the brainstem and the ANS could have a role as a “ignition‐key” for consciousness appearance (and then the cortex has the role of using information, that became or not “conscious,” progressively for adaptive purposes), what characteristics of that system is fundamental for the switching on?

This second hypothesis opens a lot of research questions. It is premature for us to review some of the results presented in this paper as a support of this hypothesis because that results was found in other settings and with other goals. We think that the question we presented should be used as a simple idea or proposal for future research. Speculatively, we can try to imagine that the amount of information on the homeostasis of the body and its variation is enormous (first characteristic) and it is continuously (second characteristic) sent to the central nervous system. Considering that it is impossible that a subject became “present” to all this information as well as attentive to it, we hypothesize that in the same way the variation of this information is proportioned to the variation from a homeostasis point. This variation represents the background actor for switching on consciousness and it is determined by the relationships among parts of a system. However, *when* the information variability switch on consciousness and *what* kind of characteristics should this fluctuation have for being a switch remain two open issues. These are important questions that should be solved in the future to evolve the drafted idea, trying to plan research that is able to go beyond some intrinsic implications reported in the second part of the next paragraph.

### 6.2 Limitations and implications

Some limitations of the study that restrict the extent to which the findings can be generalized should be overall discussed. First, the aim of this article is to present some results that are important for the scientific debate on the neural areas important for consciousness generation. It may be argued that we want to give an overvalue to the subcortical or ANS activities in contrast to those of cerebral cortex but this is not true. Simply, we want to draft an alternative point of view that starts with some clinical and experimental considerations that have intrinsic limits. An example of this is that in our approach we did not compare the results found in literature with others that support the idea that consciousness is independent from the neural system (Argonov, 2012; Georgiev, 2013, 2015) because it is intrinsic to the matter (first methodological step proposed in figure n°1) for example. The second main limitations are related to the real difficult to find studies that monitoring quantitatively the brainstem nuclei or ANS activities. Most of the study presented measure these activities indirectly, due to the high risks correlated to the positioning of electrodes for online registration or the very complex relationships between sympathetic and parasympathetic systems in humans. These problems, cause that some of our draft hypothesis is based also on indirect measures of the subcortical activities that could be influenced by variables that researchers do not consider in their studies. Finally, this is not a systematic review, so the possibility to exclude from our discussion important information available in scientific literature should be taken into consideration.

General implications emerging from the above‐mentioned hypotheses could be related both to experimental studies than clinical ones. Indeed, the studies related to consciousness should take into account the possibility of an on/off mechanism in their analysis, for example, rather than the “levels” concept which implies the subgrouping for categorizing this level. Another issue is related to the idea that the consciousness could be an on/off servomechanism generated by the relationship between parts (in this case peripheral information and central nervous system) opens the way to some important implications both in psychology, neurology, neuroethics, computational neuroscience, and philosophical perspectives (e.g., computational analysis or neuroethical perspective).

For example, it could be important for subjectivity: The idea that a subject has a body, and his/her consciousness (the consciousness of that subjective entity) could be generated only by the relationships between its parts (information from the body and information from the cortex in a continuum) implies that one single part cannot have the same capacity to generate it. In other words, consciousness for that subject could be generated as a uniqueness, which is determined by the relationships between its part (included in an uniqueness), and its generation could not be reduced to one single part like cortex, which impairments may influence the information elaboration only and not consciousness generation capacity. The second implication, if the sentence above is true, is that strong artificial intelligence theories, which sustained the possibility to generate a subjective entity replicating neural circuits in silicon, could have a chance to do it only if they are able to reproduce relationships among information as one entity, and not reproducing cortex activity alone, in the hypothesis that more complex systems than those existing today became conscious.

Moreover, if we took this line of reasoning to the extreme, another implication could be related to the idea that consciousness generation is related to the term “system”: When there are two actors in the same delimited and defined time/space horizon, there is a consciousness generation capacity. Actually, we can only suppose what kind of relationships are needed between parts to generate it but the future challenge is to explain consciousness generation overall. An out of mind question was born effectively from this article: Why a single entity, without internal or external relationships (if it is possible) should be conscious?

The fact that consciousness generation could be due to information exchange among parts could be analyzed considering the nature of this servomechanism (e.g., is it an emerging mechanism? Is it an intrinsic mechanism?) and what are laws that may govern it (e.g., Tobler's first law of geography? Dynamic system?). These are the future directions that we suggest unpretentiously, hoping that researchers can develop an appropriate epistemology and technology to go beyond the knowledge available nowadays.

## CONFLICT OF INTEREST

None declared.

## Data Availability

Data sharing is not applicable to this article as no new data were created or analyzed in this study.
